# A formula to improve the reliability of optical axial length measurement in IOL power calculation

**DOI:** 10.1038/s41598-022-23665-0

**Published:** 2022-11-07

**Authors:** Maddalena De Bernardo, Ferdinando Cione, Luigi Capasso, Alessia Coppola, Nicola Rosa

**Affiliations:** 1 Eye Unit, Department of Medicine, Surgery and Dentistry, Scuola Medica Salernitana, University of Salerno, Via Salvador Allende 1, Baronissi, Salerno, Italy; 2 Corneal Transplant Unit, ASL Napoli 1, Naples, Italy

**Keywords:** Eye diseases, Eye manifestations

## Abstract

To verify the influence of axial length (AL) variations after cataract surgery in IOL power calculation. Patients underwent ophthalmic evaluation before surgery, including optical biometry with IOLMaster 500. Same exams were repeated 2 months after surgery: AL of operated eye was evaluated using two modes (pseudophakic/aphakic options). Mean Keratometry and AL changes were analyzed. Furthermore, refractive prediction error (PE) was back-calculated with Barrett Universal-II, Hoffer-Q, Holladay-1 and SRK/T formulas. To eliminate any systematic error, the mean error (ME) was zeroed-out for each formula. MEs and median absolute errors (MedAEs) of PEs were analyzed. Two-hundred-one operated eyes of 201 patients and 201 opposite eyes were evaluated. In operated eyes, mean AL difference was − 0.11 ± 0.07 mm (p < 0.001) with pseudophakic option and 0.00 ± 0.07 mm (p = 0.922) with aphakic option. There were not-statistically significant differences between MedAE of PEs calculated after zeroing-out the ME with different ALs (p > 0.05). Instead, only MEs of PEs obtained with postoperative ALs-pseudophakic option were not-statistically different from zero (p > 0.05). AL measurement change after cataract surgery is probably due to a systematic error in optical biometer in case of phakic eyes. A correction factor applied to preoperative AL could eliminate any systematic error in IOL power calculation without modifying the lens constant.

## Introduction

The precision of IOL power calculation after cataract surgery relies on the accurate measurement of the axial length (AL), the mean corneal power (Km) and the estimation of the effective lens position (ELP)^1^. In eyes that underwent corneal refractive surgery^2–8^ or with corneal irregularities^9,10^, these values could be unreliable. These measurements, performed before surgery, do not consider eventual surgical induced changes that could influence the surgical outcome.

Concerning eventual AL variations after cataract surgery, studies performed with ultrasound (US) measurements conclude that the changes, found after cataract surgery, were related to the different sound velocity between natural lens and IOLs. For this reason, they suggested to overcome this problem utilizing different sound velocities^11,12^. In most recent years, the same problem was detected with the optical biometers, and for this reason, different group refractive indices (GRI) were incorporated in the calculation^13–15^. In fact, some authors suggested that these changes were related to a wrong assessment of the preoperative sound speed in the cataractous lens and not to the post-operative ones^16^. In a previous study, conducted in a smaller number of patients, after cataract surgery a decrease in the AL was present utilizing the pseudophakic option, whereas no significant difference was found utilizing the so called “aphakic option”^17^.

The purpose of this study was to confirm these findings in a larger group of patients and to verify their influence in IOL power calculation accuracy, according to guidelines published by Hoffer et al.^18,19^.

## Methods

In this observational case-series study, patients that underwent uneventful cataract surgery at the University Eye Clinic, Department of Medicine, Surgery and Dentistry, “Scuola Medica Salernitana”, University of Salerno, were included. The study was consistent with the tenets of the Declaration of Helsinki. Institutional Review Board approval was obtained (full fame of Approving Committee: Cometico Campania; prot. n°16544) and written informed patient consent was obtained from each patient included in the study, after explanation of the nature and possible consequences of the investigation.

Patients with dense cataracts that did not allow the use of optical biometry, with irregular astigmatism, glaucoma or previous refractive surgery were excluded from the study.

At the time of the first eye cataract surgery, both eyes of the patients underwent a complete ophthalmic examination. For the evaluation of the Km and AL, an IOLMaster 500 (5.4.4.0006; Carl Zeiss Meditec AG; website: http://zeiss.com) was utilized. The same evaluations were repeated in both eyes at least 2 months after the surgery.

For each patient, an operated eye was used for testing and the opposite unoperated eye was used as a control. Based on these criteria, 402 eyes of 201 patients (100 males) with a mean age 73.39 years and a standard deviation (SD) 7.53 years (range 50–84 years) were identified.

All patients underwent standard phacoemulsification surgery, with the implantation of a Tecnis PCB00 IOL (Abbot Medical Optics, USA; website: http://jnj.com/tag/abbott) in capsular bag.

Before and 2 months after surgery, Km and AL measurements obtained in the operated eyes were compared to the fellow eyes’ measurements obtained at the same time interval.

After cataract surgery, the postoperative AL of the operated eye was evaluated using two different options of AL measurement available on IOLMaster 500 with selecting the corresponding mode from the AL settings menu: pseudophakic option, acrylate (Ps) and aphakic option (Ap). These three options do not differ from using different GRIs, that were the same, but only in the algorithm of AL computation^14,20^, as reported in discussion section.

A stable postoperative refraction, firstly determined with an objective method (autorefractometer) and subsequently perfected with a subjective method, was identified in 133 eyes of 133 patients (63 males). The testing distance for visual acuity was 6 m and all these patients had a postoperative corrected distance visual acuity > 20/40^19^.

To obtain the refractive prediction errors (PEs) data, the Excel software (Microsoft Corporation, Redmond, WA, USA) was utilized. For each patient, different AL measurement options, preoperative keratometry readings and postoperative refraction data were inserted in the spreadsheet together with A-constant, model, and refractive power of implanted IOL. Therefore, predicted refractive outcome were retrospectively calculated using five different formulas:

- The Barrett Universal II (BUII) Formula, an updated version of Barrett Universal formula, introduced in 2010 by Graham D Barrett^21^;
- The Hoffer Q formula^22^;
- The Holladay 1 formula^1^;
- The SRK/T formula^23^;
- A combination of Hoffer Q Formula (when AL < 23.00 mm) and SRK/T Formula (when AL ≥ 23.00 mm), according to Aristodemou et al.^24^ study and hereinafter referred to as “Combo Formula”.

The last 4 formulas were programmed into our database. A random sample of 15 cases was cross-checked against the validated programming on the IOLMaster 500 to ensure consistency of output with the spreadsheet. Since BUII formula is still unpublished and the only way to calculate the IOL Power calculation with this method is through the Asia–Pacific Association of Cataract & Refractive Surgeon (APACRS) Online IOL Calculator (available at https://www.apacrs.org), predicted refractive errors of this formula were calculated trough the APACRS Online IOL Calculator.

The difference between the postoperative refraction and the preoperative predicted refraction gave the PE for each patient and for each formula. The preoperative predicted refraction was retrospectively calculated with the implanted IOL by each method. For use in IOL power calculation methods other than the SRK/T based formulas, A-constants were converted using standard relations.

To zero-out the ME with Hoffer Q, Holladay 1, SRK/T and the previously described Combo methods, the PEs were averaged and zeroed-out by applying the “Goal seek” option for the "What if analysis" function in Excel for each formula. To zero-out the ME with BUII formula, a specific computer programming language was utilized (Python, Version 3.9.3, Python Software Foundation. Available at https://www.python.org). The Median absolute error (MedAE) was calculated for all IOL power calculation methods.

Statistical analysis was performed with SPSS 26.0 (SPSS, Inc., Chicago, IL). The normality of data was examined by the exact Kolmogorov–Smirnov test. To screen whether the ME obtained by each formula was significantly different from zero, one-sample T-test was used. The Student T-test was used for pair-wise comparisons of the preoperative and postoperative AL measurements and Km values. This latter test was also used for pair-wise comparison of ME of PEs obtained by each formula with different AL measurements. For pair-wise comparison of absolute errors, the nonparametric Friedman’s test with Bonferroni correction was used.

A p value less than 0.05 was considered statistically significant. Therefore, Bland–Altman evaluation, and R^2^ analysis were performed.

Required sample size was calculated with a power calculation software (G*Power, Version 3.1.9.6, Faul, Erdfelder, Lang, & Buchner, 2020. Available at https://www.gpower.hhu.de). It was estimated that with a significance level of 5% and a test power of 80%, a sample size of 128 eyes would be necessary to detect a difference in mean error of 0.02D, given a within-subject SD for PE equal to 0.08D.

## Results

Both Km and AL data were normally distributed (all p > 0.050). There was not statistically significant difference between the Km of the operated eye and the fellow one before surgery (p = 0.700) and the AL of the operated eye and the fellow one before surgery (p = 0.656).

**Figure 1:**
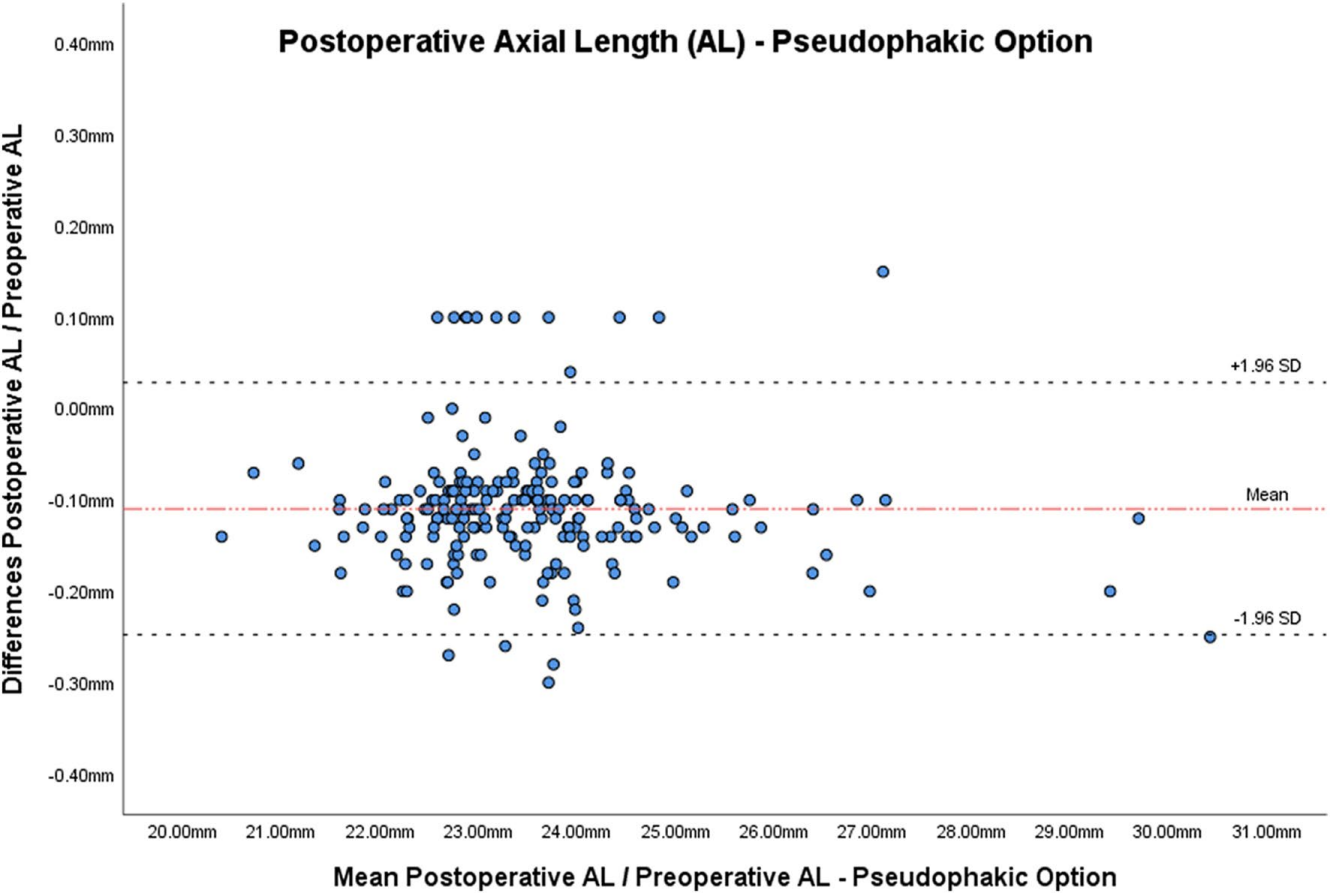
Bland–Altman plot of axial length measured before and after cataract surgery utilizing the pseudophakic option.

**Figure 2:**
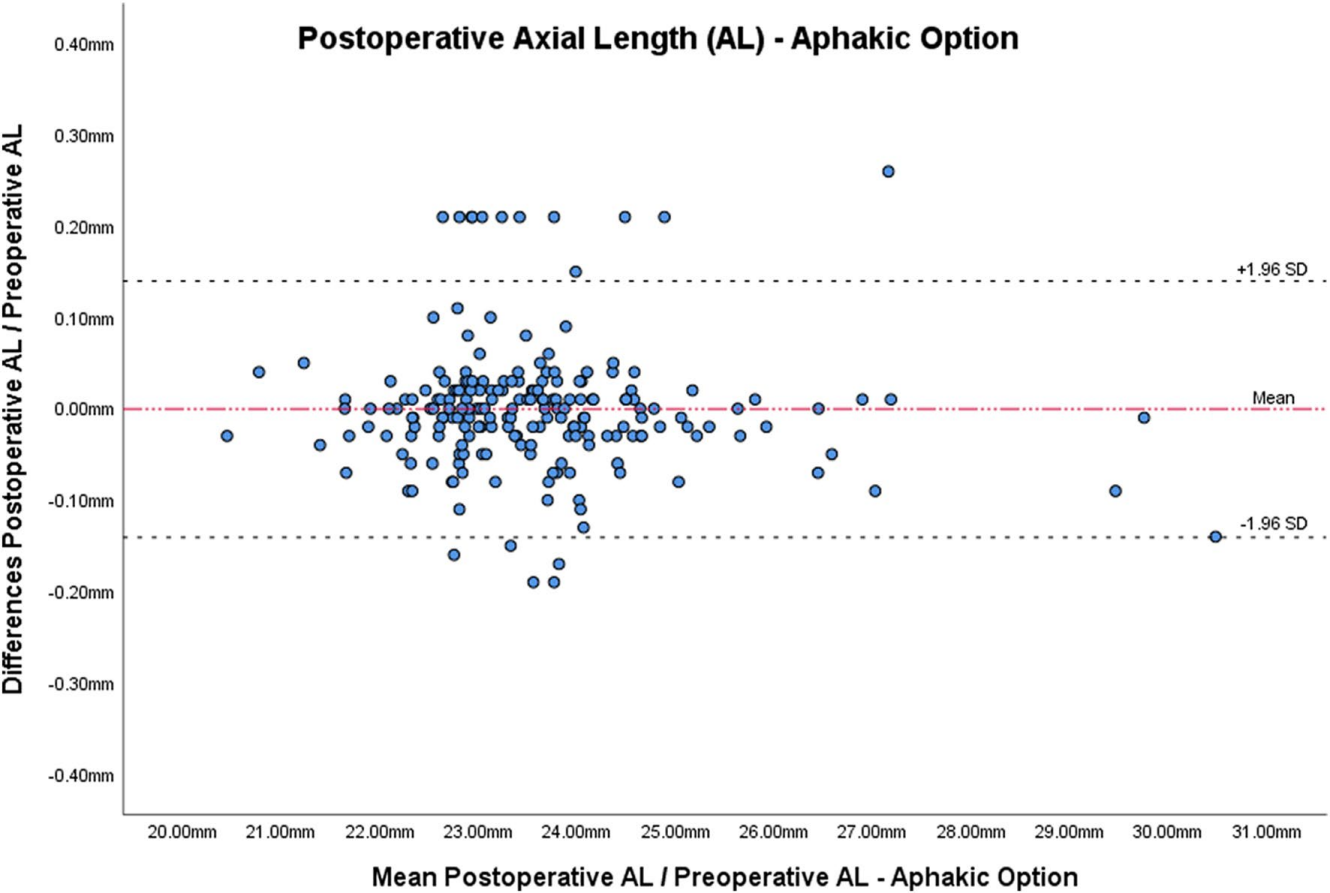
Bland–Altman plot of axial length measured before and after cataract surgery utilizing the aphakic option.

Table 1 and Figs. 1 and 2 report AL and Km in both eyes before and at least 2 months after the first eye surgery.

**Table 1:** Mean keratometry and axial length values before and after the cataract surgery.

Parameters	Before surgery		After surgery		p*
	Mean (SD)^†^	Range	Mean (SD)^†^	Range	
Km^‡^—operated eye	43.98 (1.56)D	40.10–48.12D	44.01 (1.56)D	39.94–48.29D	0.530
Km^‡^—fellow eye	44.06 (1.53)D	39.94–48.36D	44.09 (1.56)D	40.25–49.03D	0.246
AL^§^—operated eye	23.59 (1.35) mm	20.46–30.55 mm	23.48 (1.35) mm (Pseudophakic Option)	20.32–30.30 mm	< 0.001
			23.59 (1.35) mm (Aphakic Option)	20.43–30.41 mm	0.922
AL^§^—fellow eye	23.53 (1.36) mm	21.44–31.85 mm	23.54 (1.37) mm	21.44–32.17 mm	0.185

^†^Standard deviation; *p value obtained by paired T-test; ^‡^mean Keratometry, ^§^axial length.

Concerning the differences in Km, in the operated eyes (201 eyes) there was a nonsignificant increase (mean (SD) 0.17D (0.39D), p = 0.530); in the non-operated eyes (201 eyes) there was a nonsignificant increase (mean (SD) 0.03D (0.36D), p = 0.246).

Concerning the differences in AL, in the operated eyes, after surgery with the Ps, there was statistically a significant decrease (mean (SD) − 0.11D (0.07 mm); p < 0.001). With the Ap there was non-statistically significant difference (mean (SD) 0.00 mm (0.07 mm), p = 0.922). In the non-operated eyes, there was non-statistically significant difference in AL between pre- and post-surgery (mean (SD) 0.00 mm (0.05 mm), p = 0.185).

**Figure 3:**
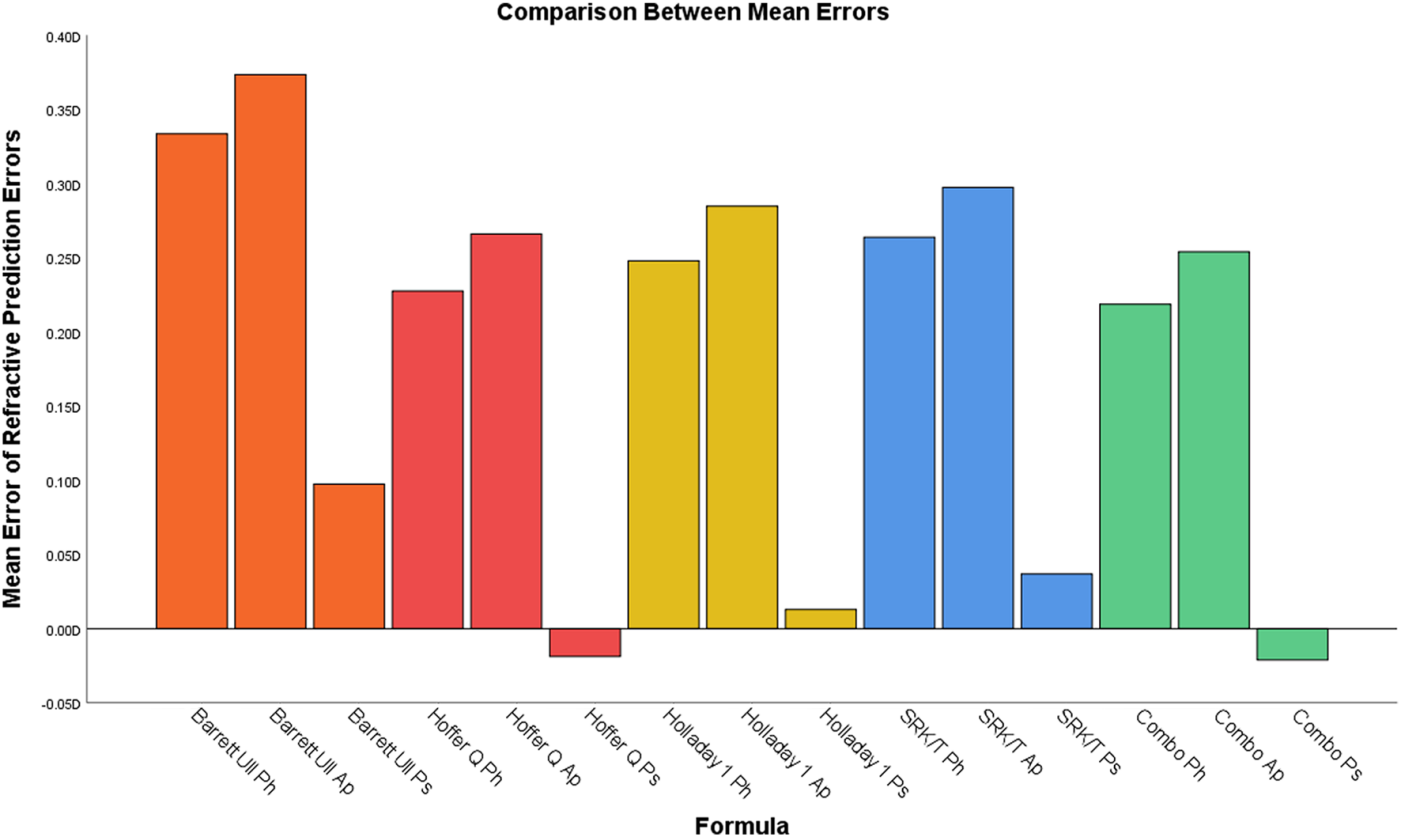
IOL power calculation formulas evaluation by utilizing phakic-option (Ph), aphakic-option (Ap) and pseudophakic-option (Ps).

All refractive PEs were normally distributed (p > 0.050). MEs of refractive PEs obtained by each IOL power calculation formula with preoperative AL with phakic option, postoperative AL-Ap and postoperative AL-Ps were reported in Table 2 and in Fig. 3.

**Table 2:** Comparison between mean errors of refractive prediction errors with ULIB constants and median absolute errors of refractive prediction errors with optimized constants for each formula.

Formula	Option	Ulib constants		Optimized constants		
		ME(SD)^¶^	P1*	ME(SD)^¶^	MedAE^††^	P2**
Barrett UII formula	Ph^†^	0.33 (0.56)D	< 0.001	0.00 (0.58)D	0.34D	Ph vs Ps > 0.05
	Ps^‡^	0.09 (0.59)D	> 0.05	0.00 (0.60)D	0.32D	Ps vs Ap > 0.05
	Ap^§^	0.37 (0.62)D	< 0.001	0.00 (0.64)D	0.33D	Ph vs Ap > 0.05
Hoffer Q formula	Ph^†^	0.23 (0.65)D	< 0.001	0.00 (0.67)D	0.41D	Ph vs Ps > 0.05
	Ps^‡^	− 0.02 (0.68)D	> 0.05	0.00 (0.68)D	0.35D	Ps vs Ap = 0.025
	Ap^§^	0.27 (0.70)D	< 0.001	0.00 (0.73)D	0.37D	Ph vs Ap > 0.05
Holladay formula	Ph^†^	0.25 (0.60)D	< 0.001	0.00 (0.62)D	0.30D	Ph vs Ps > 0.05
	Ps^‡^	0.01 (0.63)D	> 0.05	0.00 (0.63)D	0.34D	Ps vs Ap > 0.05
	Ap^§^	0.28 (0.65)D	< 0.001	0.00 (0.68)D	0.33D	Ph vs Ap > 0.05
SRKT formula	Ph^†^	0.26 (0.57)D	< 0.001	0.00 (0.58)D	0.35D	Ph vs Ps > 0.05
	Ps^‡^	0.04 (0.59)D	> 0.05	0.00 (0.59)D	0.33D	Ps vs Ap > 0.05
	Ap^§^	0.30 (0.62)D	< 0.001	0.00 (0.63)D	0.35D	Ph vs Ap > 0.05
Combo formula	Ph^†^	0.22 (0.61)D	< 0.001	0.00 (0.61)D	0.35D	Ph vs Ps > 0.05
	Ps^‡^	− 0.02 (0.65)D	> 0.05	0.00 (0.63)D	0.33D	Ps vs Ap > 0.05
	Ap^§^	0.25 (0.66)D	< 0.001	0.00 (0.65)D	0.35D	Ph vs Ap > 0.05

^†^Preoperative axial length, phakic option; ^‡^postoperative axial length, pseudophakic option; ^§^postoperative axial length, aphakic option; ^¶^mean error (standard deviation); *p value obtained by one-sample T-test to check whether the value was different from zero; ^††^median absolute error; **p value obtained from pair-wise comparison of absolute errors by Friedman’s test with Bonferroni correction.

For each formula, there were statistically significant differences between MEs of PEs obtained by utilizing the preoperative AL and by utilizing the postoperative AL-Ps. These differences were noted with BUII, Hoffer Q, Holladay 1, SRK/T and Combo formulas (all p < 0.001). On the other hand, there were not statistically significant differences between ME obtained by utilizing the preoperative AL and by utilizing the postoperative AL-Ap in all formula analyzed (all p > 0.050).

To check whether MEs obtained from all formula analyzed were significantly different form zero, one-sample T-test was performed. In all the cases, MEs for all IOL power calculation methods utilizing postoperative AL-Ps were not statistically different from zero (all p > 0.050), as shown in Table 2. In all other cases, the MEs were statistically different from zero (all p < 0.001). Optimized lens constants after zeroing out all the MEs were reported in Table 3.

**Table 3:** Optimized constants after zeroing out the mean error with different axial length measurements for each formula.

IOL model		Ulib lens constant
Tecnis PCB00 IOL (Abbot Medical Optics, USA)		A-Constant = 119.30 pACD^¶^ = 5.80 SF^††^ = 2.02
Formula	Option	Optimized lens constant
Barrett UII formula	Ph^†^	A-Constant = 119.73
	Ps^‡^	A-Constant = 119.44
	Ap^§^	A-Constant = 119.73
Hoffer Q formula	Ph^†^	pACD^¶^ = 5.97
	Ps^‡^	pACD^¶^ = 5.79
	Ap^§^	pACD^¶^ = 6.00
Holladay formula	Ph^†^	SF^††^ = 2.20
	Ps^‡^	SF^††^ = 2.03
	Ap^§^	SF^††^ = 2.22
SRKT formula	Ph^†^	A-Constant = 119.60
	Ps^‡^	A-Constant = 119.34
	Ap^§^	A-Constant = 119.64
Combo formula	Ph^†^	A-Constant = 119.61 / pACD^¶^ = 5.91
	Ps^‡^	A-Constant = 119.34 / pACD^¶^ = 5.75
	Ap^§^	A-Constant = 119.65 / pACD^¶^ = 5.93

^†^Preoperative axial length, phakic option; ^‡^postoperative axial length, pseudophakic option; ^§^postoperative axial length, aphakic option; ^¶^predicted anterior chamber depth; ^††^surgeon factor.

All MedAE obtained following to lens constants optimization for each formula, utilizing preoperative AL, postoperative AL-Ps and postoperative AL-Ap, were reported in Table 2. According to Friedman’s test with Bonferroni correction, there were not statistically significant differences between values for each formula analyzed (all p > 0.050,) except for results obtained between Hoffer Q formula utilizing postoperative AL-Ps and postoperative AL-Ap (p = 0.025).

## Discussion

To decide the IOL power to be inserted in the eye in case of cataract surgery, precise preoperative Km and AL measurements are necessary.

For a long time, US has been considered the gold standard in AL measurement. Today the introduction of optical devices made these instruments the new gold standard^13^. Utilizing these new tools, not only after photorefractive keratectomy^8^, but also in case of cataract surgery^17^, a slight but significant reduction in the AL measurement have been shown.

Different hypotheses have been suggested to explain the AL changes:

1. A reason could be a lack of repeatability in the IOLMaster 500 measurements, but the presence of no significant differences in the fellow non-operated eyes makes this hypothesis too unrealistic.
2. A Km decrease (or better, a corneal radius increase) after surgery that could flatten the anterior chamber with a subsequently AL reduction was proposed^17^ but, in the present study as in previous ones^17^, no significant changes in the Km values were found, making this hypothesis impracticable.
3. It was possible to hypothesize that the lens extraction causes a decrease in the eye volume, with subsequent AL decrease, but no significant shortening of the eye after cataract surgery was found in a large series^11^.
4. There are no real changes. The use of different propagation speeds to improve the AL measurement results were suggested^12^ and the same criteria were utilized when partial coherence interferometry (PCI) was applied in the IOL power calculation^13^, and a subsequent study suggested that to have equivalent pre- and post-operative measurements, a 0.12 mm corrective factor for the acrylic and 0.08 mm for the polymethylmethacrylate lenses should be employed^14,20^. This correcting factor is utilized by the IOLMaster 500, which adds 0.1 mm in case of pseudophakic measurement. In the present study it was found that even with this correcting factor, a decrease in the AL is still present. Either a real decrease in the AL measurement or an inaccuracy in above-mentioned correcting factors, that could not be sufficient to correct the AL measurement, could explain such decrease^14^. The inaccuracy of these correcting factors could be related to the fact that AL measurement with the IOLMaster 500 was compared to contact A-scan ultrasound technique in the cited study^14^. Today it is well known that A-scan immersion and not contact technique should have been used for comparison.
5. The wrong measurement is the preoperative one, because the real refractive index of the implanted lens is known, whereas the refractive index of the human lens could change according to the cataract grade. The last hypothesis is supported by Drexler et al.^16^ who found that the difference in pre- and post-operative AL was compensated by changing the preoperative lens refractive index.

Regarding refractive index discussion, since IOLMaster 500 uses PCI technology, that cannot measure Lens Thickness (LT), it was not possible to use the specific refractive index of each intraocular dioptric medium and a GRI was used. Because of different components of the eye are more variable (e.g., thicker lens, deeper anterior chamber), GRI may be suboptimal^25,26^.

Many authors questioned the validity of traditional AL measurements by optical biometry and investigated the so-called “sum-of-segments AL”. Segmented AL is today available on the Argos SS-OCT (Movu, Inc., Komaki, Japan) and it was used by Shammas et al.^26^ to compare this AL to traditional AL measured with a single refractive index. They found statistically significant differences between ALs, with a traditional AL shorter in short eyes and longer in long eyes. Savini et al.^27^ described how also an SS-OCT optical biometer based on the GRI (IOLMaster 700; Carl Zeiss Meditec, AG) overestimates AL compared to the device using segmented AL in long eyes (Argos). The effect of these differences on IOL power calculation were not evaluated in both studies.

Both Wang et al.^28^ and Cooke et al.^29^ found that traditional AL was longer for long eyes, shorter for short eyes, and about the same for medium-length eyes.

Wang et al. reported that by using the segmented ALs PEs were improved in short eyes with the Hoffer Q and Holladay 1 formulas and in long eyes with BUII, Haigis, Hoffer Q, Holladay 1 and SRK/T formulas^28^. Cooke et al. reported that by using segmented AL in both short and long eyes PEs were improved in formulas designed on US data, although it worsened in optical-biometry-derived formulas, such as the BUII formula^29^.

Because of refractive index of the human lens could be unreliable, this study proposes an alternative approach to the sum-of-segments AL, taking into consideration the postoperative AL and its relationship with PEs, not analyzed in previous studies.

The reliability of the AL measurement was therefore evaluated with the analysis refractive PE determined in the IOL power calculation, using all formulas included in the IOLMaster 500 biometer, except for Haigis formula^13^, because it requires the knowledge of Anterior Chamber Depth (ACD), that in pseudophakic eyes is unreliable: therefore, calculation biases could occur, even if preoperative values ​​were used. In addition, BUII formula, not included in the IOLMaster 500, was analyzed. Although ACD knowledge was recommended also for BUII formula, together with other optional parameters, it is possible to use this formula even without this information^30,31^. On the other hand, it was not possible to include other fourth-generation IOL power calculation formulas due to the necessity to know the ACD measurements to work reliably^32,33^.

With all analyzed formulas, as shown in Table 2, the use of the postoperative AL-Ps to back-calculate the refractive PE, reduces the influence of the systematic error deriving from the optical biometer^19^. In fact, in all these cases, the calculated ME was not statistically significant different from zero, eliminating in this way the necessity to zero-out the ME.

On this basis, a linear regression formula was identified to correct the preoperative AL, to eliminate any systematic error deriving from the biometer, according to Hoffer et al.^18,19^ protocols:

$$\mathrm{ALc}= -0.017 + 0.996*\mathrm{AL}$$

where ALc is the corrected AL and AL is the preoperative AL detected with optical biometer by utilizing phakic option.

This regression formula could be additionally useful in studies that analyze IOL power calculation formula accuracy, because it eliminates any systematic error without changing the lens constant trough zeroing-out the ME. In fact, the use of ALc already made the ME equal to zero. Furthermore, this correction factor could be essential in analyzing IOL power calculation formulas accuracy, because zeroing out the ME could be difficult with standard methods in some cases, for example when evaluating unpublished formulas^19^.

This study has several limitations. Wang-Koch (WK) adjustments of AL for Holladay 1 and SRK/T formula in long eyes were not applied^34,35^. However, only 4 eyes of 133 analyzed eyes with a stable postoperative refraction had AL > 26.5 mm and updated WK AL adjustments^35^ should be applied when AL > 26.5 mm for Holladay 1 formula or when AL > 27.0 mm for SRK/T formula. In addition, constants optimization trough zeroing out the ME was performed for all formulas: new lens factors were all different from ULIB lens factors and some bias could be generated if optimized constants were used together with WK AL adjustments, that are projected for ULIB lens factor^35^. Another limitation of this study is that it was conducted with the IOLMaster 500 biometer, but considering the identification of a similar alteration in AL found before and after cataract extraction using the IOLMaster 700^36^ and the good correlation between the measurement of AL with IOLMaster 500 and other optical biometers, it is possible to hypothesize the use of the correction factor of the preoperative AL similarly in other biometers^37–40^. In fact, in most recent studies regarding AL measurement comparison with different optical biometer, only small differences, often statistically significant but always < 0.03 mm, have been detected between IOLMaster 500 and other biometers^37–40^. Further studies are needed in this regard. In addition, based on the similar results obtained even with BUII formula, a new generation formula not included in the IOLMaster 500, it is possible to theorize the application of ALc additionally in other newest IOL power calculation formulas.

It is necessary to perform further studies on different and larger samples, with the inclusion of other newer generation IOL power calculation formulas not included in this paper^32,33^, to verify the effectiveness of the ALc.

On the other hand, the presence of a single IOL model might seem a limitation of this study, but it is fundamental to zero-out the ME and then to evaluate the accuracy of the IOL calculation formulas.

It cannot be said whether ALc should be considered the "true" AL, but it certainly represented the most accurate value to be included in the IOL calculation formulas because it can eliminate any systematic error, as described above. According to the results of the present study, after cataract surgery there was a reduction in AL measurement with pseudophakic option, that was not detected with the aphakic option. IOL power calculation with postoperative AL-Ps resulted in a ME equal to zero (Fig. 3): in this way the systematic errors in the measurement of AL with optical biometer was eliminated. The result of this study suggests the use of a correction factor applied to the AL measurement obtained before cataract surgery to optimize preoperative AL measurement, with the elimination of any systematic error, without modifying the lens constant.

## Data Availability

The datasets used and analysed during the current study available from the corresponding author on reasonable request.
